# Habitat Fragmentation and Lichen Diversity in Peri-Urban Woodlands: A Case Study in the Municipality of Potenza (Southern Italy)

**DOI:** 10.3390/plants11141858

**Published:** 2022-07-15

**Authors:** Giovanna Potenza, Gianluca Gerardi, Simonetta Fascetti, Leonardo Rosati

**Affiliations:** School of Agricultural, Forest, Food and Environmental Sciences, University of Basilicata, 85100 Potenza, Italy; gianluca-gerardi@libero.it (G.G.); simonetta.fascetti@unibas.it (S.F.); leonardo.rosati@unibas.it (L.R.)

**Keywords:** air pollution, epiphytic lichens, landscape ecology, Mediterranean ecosystems, Quercus pubescens forests, species richness

## Abstract

The fragmentation of the natural habitat is a process that is exponentially increasing worldwide and represents one of the biggest threats to biological diversity. Habitat destruction and fragmentation have a major impact on landscapes and may also affect ecosystems, populations, and species. The ongoing anthropogenic process can result in habitat loss for some species, habitat creation for others, reduced patch size, and increased distance between patches, which may lead to local extinction. We analyzed the effects of patch size and isolation on lichens in *Quercus pubescens* woods surrounding the city of Potenza (south Italy). We randomly sampled 11 forest patches with homogeneous environmental variables using circular plots with a 10 m radius; the patches ranged from 0.3 to 30 ha. For each plot, we collected data about presence and abundance of epiphytic lichens. We performed the analyses at the patch level using linear regression and multivariate analysis, searching for effects on species richness, life forms, and community compositions. Multivariate analyses were used to study the effect of fragmentation on the structure of lichen vegetation. We investigated the main predictor of lichen species richness in habitat fragmentations and concluded that patch area per se is an important (positive) driver of lichen species richness in Mediterranean peri-urban forests.

## 1. Introduction

The last decades have been characterized by increased migration from rural to urban areas [1], especially in developing countries. For the first time since 2008, more than half of the world’s population lives in towns and cities, and this percentage is expected to increase to 70% by 2050 [2].

Urban growth is able to modify landscapes and the related patterns of biodiversity of urban ecosystems. Moreover, the ecological footprint of cities influences the surrounding natural landscape and rural urban interface [3].

Urban areas are highly modified and complex landscapes, but green or open areas are seen as valuable for human beings as well as for wild species [4,5].

The relationship between the conservation of biodiversity and urban areas clearly emerged at the end of the 20th century and was recognized in 1993 in the framework of the Convention of Biological Diversity (CBD). Despite urban areas representing only about 3% of the global land cover [6], their expansion has caused a huge transformation of habitat and landscape; at present, they represent a major threat to biodiversity conservation for several countries [7,8].

Cities are not randomly located on a global scale but are often positioned in biodiversity hotspots. Because the population is growing at an extraordinary pace, especially in developing countries, conflicts with conservation goals are increasing [9,10,11,12,13,14].

The urban forests of a city can represent a strategical network of high-quality natural areas that are designed and managed to deliver a wide range of ecosystem services and to protect biodiversity in urban and peri-urban settings.

Many studies [8,15] have reported the necessity of preventing biodiversity losses in (semi)natural landscapes that are affected by urban or agriculture growth and environmental pollution produced by human activities (e.g., air pollutants).

The protection and restoration of urban forests can help create a healthy environment, even though such forests often are appreciated more for their aesthetic value than for their ecosystem functions. Nevertheless, urban forests help to create and enhance habitats constituting a pool of biodiversity, significantly improving soil quality and contributing to land restoration and mitigation of the environmental pollutants produced by cities. In particular, urban woodlands may help preserve and increase biodiversity [16,17].

This study focused on epiphytic lichen biodiversity patterns related to fragmentation within urban woodlands; habitat fragmentation in urban woodlands is recognizably considered one of the most important threats to biodiversity. For example, several studies have confirmed that generally smaller fragments with a large perimeter-to-area ratio tend to be far from each other and have a small variety of microhabitats; moreover, they are more vulnerable to the negative effects of habitat fragmentation and are inefficient for biodiversity conservation [18,19,20,21].

Theoretically, habitat fragmentation alters plant community dynamics by influencing both local (within patch) and regional (among patch) processes. Fragmentation increases local extinction risk by lowering mean population sizes [22], reducing habitat (area effects), altering border environments (edge effects), and, at later stages, depressing immigration potential (isolation effects) [23,24,25,26,27].

Lichens may be more sensitive or may more rapidly respond to the effects of forest degradation and fragmentation, acting as foretellers of posterior synergetic effects. Due to their significant sensitivity to the inevitable microclimatic alterations resulting from habitat loss and degradation, they may be particularly useful as early indicators of the adverse effects of forest fragmentation.

According to the “rescue effect” hypothesis, habitat destruction, which undermines the distribution of several forests, will eventually result in synchronized reduction in both species occurrence and abundance, as a consequence of lower “per-patch” rates of colonization.

To test this hypothesis, previous experimental research, searching for the effects of habitat fragmentation on communities and natural landscape, was only performed at the micro-scale level.

However, many of these experiments limited the spatial or temporal scale of the studies of microsystems by focusing on taxa whose habitat patches are not entirely related to the analyzed fragments and might have included confounding effects such as inaccurate area, age, and isolation [26,28,29,30], or by focusing on communities including taxa whose habitat patches are not entirely restricted to the study fragments [31].

Three major threats are noted for their potential to alter lichen diversity: forest management, air pollution, and climate change [32,33,34,35,36,37]. Lichens are more sensitive to land management; thus, they are important indicators of forests that are close to natural conditions [38].

Epiphytic lichen diversity is also related to forest structure and dynamics, e.g., [39,40,41,42,43]. Several environmental factors are relevant to the dispersal, establishment, and maintenance of epiphytic lichens that are also affected by forest management, e.g., [41,44].

In our study, we focused on species richness and community composition, because biodiversity loss is not restricted to rare species but has also been reported in common species. In order to assess if habitat fragmentation affects the diversity and composition of epiphytic lichens, we investigated epiphytic lichens in a group of 11 forest fragments within a matrix of agricultural land residues recently affected by the urbanization process in the peri-urban belt of the city of Potenza in the Basilicata region of south Italy.

## 2. Materials and Methods

### 2.1. Study Area

**Figure 1:**
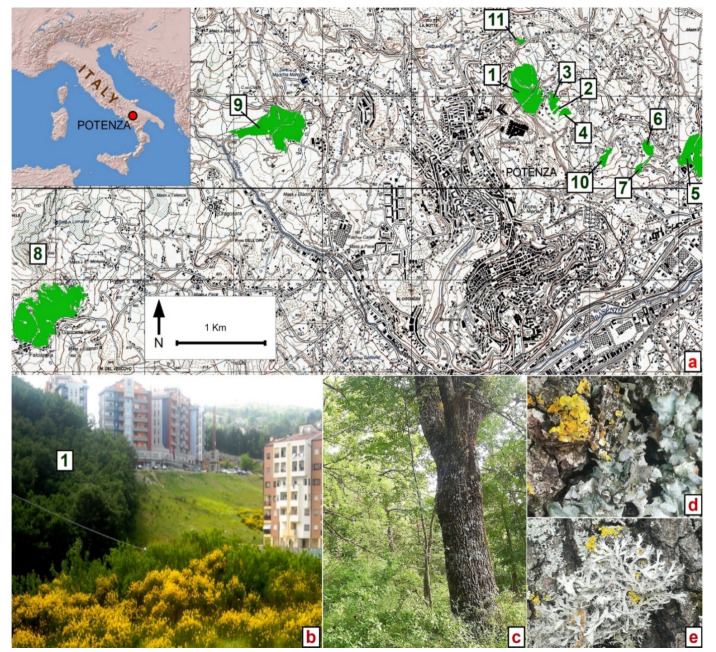
(**a**) Study area location: green patches are the 11 peri-urban *Quercus pubescens* woodlands sampled to assess their epiphytic lichens diversity and composition; forest patch numbers correspond to those in Table 1; (**b**) new urban areas in the periphery of Potenza city (Macchia Romana) close to the forest patch number “1” with flowering of *Spartium junceum* shrubs, which are recolonizing uncultivated areas; (**c**) *Quercus pubescens* tree at Macchia Giocoli (patch n. 8); (**d**) *Pleurosticta acetabulum* (Neck.) Elix & Lumbsch and *Xanthoria parietina* (L.) Th. Fr. on *Q. pubescens*; (**e**) *Anaptychia ciliaris* (L.) Flot, *Physcia adscendens* (Fr.) H.Olivier, and *Xanthoria parietina* (L.) Th. Fr. on *Q. pubescens* bark.

The study was conducted in the peri-urban woodlands of the city of Potenza in Basilicata Region (Figure 1), situated in the Lucanian Apennines of south Italy.

The topography is shaped by hilly reliefs on sandy-clay substrata, at an altitude between 700 and 1000 m a.s.l. The climate in this area is meso-temperate, humid–subhumid, with a sub-Mediterranean feature (a dry season occurs during the summer); the mean annual temperature is 12.8 °C and the mean annual rainfall is 650 mm [45].

The natural vegetation of this territory on sandy soil, generally close to hilltops, is characterized by thermophile oak forest of *Centaureo centauri–Quercetum pubescentis* Zanotti, Ubaldi, Corbetta & Pirone, while the mesophile areas on deeper clay soil are dominated by *Quercus cerris* communities [46]. The current land use is dominated by urban and agricultural areas, with only few remnants of oak forests.

### 2.2. Sampling Design

Peri-urban woodlands of the municipality of Potenza were mapped from ortho-rectified high-resolution aerial digital images and selected using the following criteria:

i. Remnants of oak forests dominated by *Quercus pubescens* (*Centaureo centauri-Quercetum pubescentis*);
ii. Homogeneous environmental variables (climate and geomorphology);
iii. Accessibility (some small patches of fenced woods were excluded).

The oak forests were identified as the object of this study because they represent the dominant potential natural vegetation of the surroundings of the city of Potenza, and, in particular, the stands dominated by *Quercus pubescens* represent the almost exclusive typology of remnant forest fragments; in addition, they host one of the most abundant residual populations of *Rhaponticoides centaurium* (L.) M.V.Agab. & Greuter (= *Centaurea centaurium* L.), a rare nemoral vascular plant, endemic to the southern Apennines.

Based on a stratified random sampling, 11 patches and 11 random circular plots of 10 m radius were selected and surveyed (Table 1 and Figure 1).

To limit the edge effect, a buffer zone (10 m width) was excluded within each selected patch.

**Table 1:** Coordinates of sampling plots with environmental variables, forest structure parameters, and landscape variables of each sampled woody patch. Patch IDs correspond to those in Figure 1.

Patch Id	X (UTM 33 WGS 84)	Y (UTM 33 WGS84)	Aspect (Degree)	Slope (Degree)	Altitude (m a.s.l.)	No. Trees (>5 cm)	Basal Area (m^2^)	Area (ha)	Perimeter (m)	Species Richness
1	568125	4501074	255	10	810	107	0.93	9.0744	3269	21
2	568567	4500668	255	10	775	71	0.96	0.3264	293	19
3	568534	4500765	315	15	780	74	0.5	1.4299	888	16
4	568666	4500638	248	45	770	75	0.88	0.7866	557	21
5	570061	4500342	135	25	740	18	0.75	8.2519	2847	29
6	569596	4500212	248	20	760	61	0.92	1.5052	619	20
7	569471	4500031	270	35	720	63	0.92	0.8432	926	21
8	562809	4498301	90	30	990	24	0.76	30.7503	8755	26
9	565562	4500561	225	10	820	43	0.63	18.1794	5354	30
10	569072	4500100	270	10	750	43	0.92	1.3865	903	19
11	568139	4501416	270	25	820	86	0.9	0.4854	377	21

### 2.3. Lichen Sampling

From April to August 2012, epiphytic lichen species found on the trunk of the 3 trees nearest to the center of each circular 10 m diameter random plot were sampled within each selected forest patch. Trees with diameter at breast height (DBH) >16 cm and trunk inclination <30 °C were considered suitable for lichen data collection [47]. A total of 33 trees were surveyed in the study area. The number and relative abundance of lichen species were determined for each tree up to a height of 2 m. Lichens growing on the ground and on rocks were not considered in our analysis.

Species that were difficult to identify in the field were collected for identification in the laboratory using a binocular Nikon (Minato, Japan) Model C-PS (magnification up to ×40). For section of thalli and fruiting bodies, a polarized light microscope (Nikon YS100), with ×4, ×10, ×40, and ×100 objectives with the possibility of oil immersion was used. Chemical spot tests or K (10% aqueous), C (saturated aqueous bleach), KC (combination), and Pd (5% alcoholic p-phenylenediammine) were performed when necessary.

Species nomenclature, life forms, the type of symbiosis, and ecological indicator values follow [48] and are continuously updated in the online database ITALIC (https://italic.units.it/index.php accessed 20 February 2022).

Specimens were deposited in the Herbarium Lucanum (HLUC) hosted at Basilicata University (Potenza, Italy).

### 2.4. Data Analysis

Species richness was calculated as the number of species per plot. Linear regression analysis was used to test the significant relationship between species richness and the different group of variables regarding landscape (patch area, isolation, shape index, and distance from city center), environment (altitude, slope, and aspect) and forest structure (tree density, basal area, tree height, tree layer cover, and trunk diameter). Due to lack of fine-scale data regarding air quality in the studied area, the distance from the city center was considered as a proxy for air pollutant concentrations because it is mainly due to vehicular traffic and building heating.

Finally, a multivariate analysis (PCoA; Bray-Curtis distance) was performed to explore the combined effect of the landscape, environmental, and forest structure variables on the structure and composition of lichen vegetation as a whole. Retained variables were standardized and superimposed to the ordination axes of PCoA. Aspect (slope exposure) was converted to radians and then to a southing index S with the formula S = −cos (aspect) + 1. Statistical analyses were performed using PAST 4.10 and PRIMER 6.1.11.

## 3. Results

### Species Richness and Composition

A total of 37 epiphytic lichen taxa were found (Table 2). Among the landscape variables, isolation, shape index, and distance from the city center were not further investigated in this specific “archipelago” of forest patches as it was significantly (r^2^ > 0.65; *p* < 0.001) and positively correlated with patch area, i.e., larger patches were more isolated, less convoluted, and more distant from the city in our study area.

Moreover, a post hoc test, looking for a correlation between species richness and edge distance, was performed to ensure we properly removed the edge effect from the sampling design. As result, no significant correlation between species richness and edge distance was found (r^2^ = 0.03; *p* = 0.35).

As for forest structure, both tree height and tree density significantly corresponded to trunk diameter and basal area, respectively; thus, they were removed from the subsequent statistical analyses.

**Figure 2:**
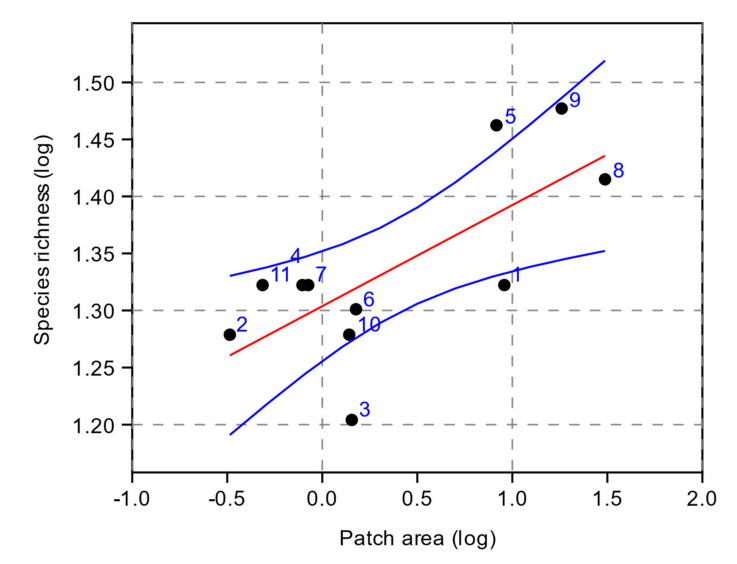
Linear model of lichen richness and patch_area relationship at plot level. The red solid line is the linear trend line; blue lines represent the 95% confidence intervals. Numbers of the plots are the same as in Table 1 and Figure 1.

Species richness resulted positively and significantly related to the patch area (r^2^ = 0.5, *p* = 0.01) of the analyzed forest fragments (Figure 2).

**Figure 3:**
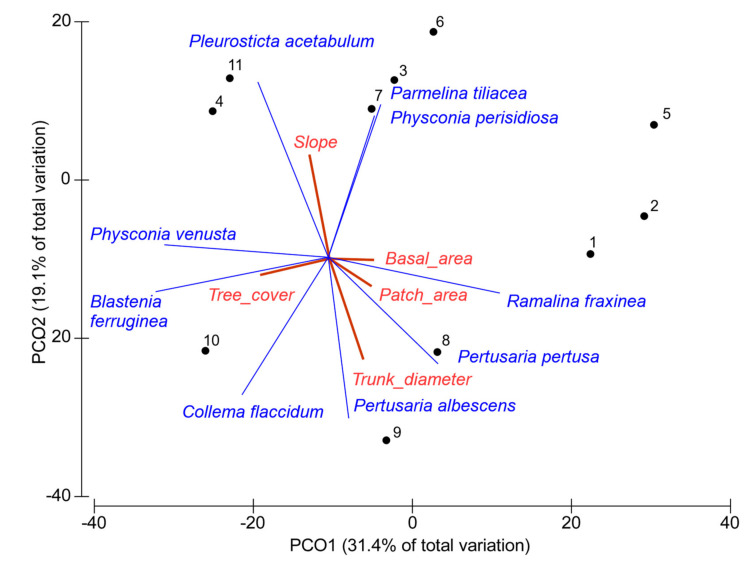
Triplot of the two first principal components from analysis of landscape, environmental, and forestry variables (patch area altitude, slope, aspect, basal area, cover tree layer, and trunk diameter). Only variables having Spearman’s correlation >0.2 are shown. Correlation’s threshold for lichen species was set at 0.7.

PCoA allowed us to determine which variables contribute the most to the differences in species composition and abundance among plots. The first two principal components accounted for 50.5% of the total variance. The loadings of the variables on the two retained PCs are presented in Table 3. Considering the lichen vegetation as a whole, the ordering axes highlighted the absence of a clear pattern in the distribution of forest fragments in relation to the analyzed variables. In particular, no evident relationship emerged between the composition of lichen vegetation and the higher species richness of the largest forest fragments revealed by univariate analysis. Larger patches (i.e., 8, 9, 1) appeared poorly grouped together even if some species (*Ramalina fraxinea*, *Pertusaria pertusa*, *P. albescens*) seemed to indicate a better state of conservation of these patches, linked to greater basal area and tree diameters. Furthermore, considering how much the abundance of epiphytic lichens is influenced by air humidity, unexpectedly, no significant correlation emerged between species richness and slope exposure (lower richness was expected in the south-facing slopes, which are generally considered to be more arid). On the contrary, slope exposure was significantly and positively correlated with species richness, one of the variables most correlated with the second ordination axis (Table 2 and Figure 3). In addition, this result can be considered contrary to the expected, when it was observed (Table 1), as, in the study area, the more inclined slopes have a prevalent west-southwest exposure; thus, the combination of these factors should emphasize aridity by increasing temperature and decreasing air humidity. These results seem to suggest that, in the study area, the microclimatic variations due to slope and aspect only have low incidence on the overall species richness at the patch level, but they can determine a shift in species composition at the community level. This shift is indicated by the species correlated with the second ordination axis shown in Figure 2. Notably, based on their ecological indicator values reported in italics, *Pleusticta acetabulum*, *Parmelina tiliacea*, and *Physconia perisidiosa* (i.e., positively related to steeper slopes) have higher aridity index values; on the contrary, *Pertusaria albescens*, *Pertusaria pertusa,* and *Collema flaccidum* (negatively related to slope values) have lower aridity index values, thus resulting in relatively hygrophytic species with respect to the former.

## 4. Discussion

Of the 37 lichen species found, 9 were crustose, 1 was leprose, 11 were foliose broad-lobed (*Parmelia*-type), 11 were foliose narrow-lobed (*Physcia*-type), and 5 were fruticose. One species, *Pectenia plumbea*, was symbiotic with filamentous cyanobacteria and one, *Collema flaccidum*, with coccaceous cyanobacteria [48]. Regarding the reproductive strategy, 20 species had a mainly sexual reproductive strategy; 13 were mainly asexual, using soredia or soredia-like structures; and 4 were mainly asexual, producing isidia, or isidia-like structures. As for poleotolerance, 14 species also occurred in heavily disturbed areas, including, for example, large towns; 18 species occur also in moderately disturbed areas (e.g., agricultural areas and small settlements); 4 species (*Lecanora albella*, *Melanohalea elegantula*, *Physconia venusta*, and *Ramalina fraxinea*) mostly occurred in natural or semi-natural habitats [47]. Notably, one species, *Pectenia plumbea,* which exclusively occurs on old trees in ancient undisturbed forests, was only found in one of the largest forest patches (two occurrences). On the other hand, five species, considered as pioneer (*Lecanora chlarotera*, *Lecidella elaeochroma*, *Melanohalea exasperata, Myriolecis hagenii*, and *Physcia adscendens)*, were detected in all plots.

The size of the forest fragment was a significant and positive predictor of species richness, in agreement with several other studies, e.g., [49,50,51] in particular with data on vascular plants’ distribution in a Mediterranean forest ecosystem [49].

From the level of species, we highlighted a strong correlation between the presence of more demanding fruticose lichens with the fragment size and some structural parameters (basal area and average diameter of the trees), in agreement with [52]. The presence of *Anaptychia ciliaris* (L.) Flot was mainly related to higher basal area values of the plot. The presence and abundance of a group of species characteristics of *Xanthorion* communities instead correlated with sites characterized by a greater slope.

The results of the multivariate analyses at the patch level (Table 3 and Figure 3) showed that the diversity of lichen flora (richness and community composition) was not particularly related to any of the analyzed environmental and forestry variables (altitude, slope, aspect, basal area, cover tree layer, or trunk diameter), according to [50]. After the removal of the edge effect between landscape variables, the only variables left (due to the autocorrelation of the others) were found to be significantly and positively correlated with species richness, but this effect was not so evident when the overall composition of the lichen vegetation (composition and abundance) was considered.

It was not possible to separate the effect of isolation and shape of the fragments because these landscape parameters strongly correlated with the size of the fragment in our case study. This finding can be explained by the particular configuration of the group of fragments group under investigation, in which the smaller fragments were the result of recent fragmentation and were therefore closer to each other. Not considering this, we would have obtained a result contrary to that expected, with a higher diversity in the isolated fragments.

The results showing that lichen diversity and community composition are not correlated with any of the measured environmental and forest structural parameters could be considered due to a certain homogeneity of these variables within the study area, indirectly supporting the validity of the performed stratified sampling, aimed at minimizing, as much as possible, the effect of these confounding factors. In particular, microclimatic factors possibly related to air humidity as slope and exposure proved to be irrelevant for species richness, but could determine some shifts in species composition. As shown by the results of multivariate analyses, the epiphytic lichen vegetation of *Centaureo centauri-Quercetum pubescentis* forest fragments is quite homogenous as a whole, and it is not evidently shaped by forest patch dimension; thus, other factors such as slope inclination, stand age (indicated by mean trunk diameter), or past management (not assessed in this study) may contribute to explaining its variation. However, the role of potential bioindicators of some of the species highlighted by ordination deserve attention in a future detailed study. Considering that lichens are extremely sensitive to changes in air pollution, the latter should be an important explanatory variable to consider in a peri-urban ecosystem. Unfortunately, high-resolution data about the air quality in Potenza are not available thus, it was not possible to relate environmental pollutants to lichen diversity and composition. Furthermore, in this study, patch area strongly correlated with the patch distance from the city center (i.e., a landscape variable, which can be assumed to be reasonably related to decreasing levels of atmospheric pollution); thus, it had to be discarded. We hope that data will be available in the future to evaluate this crucial issue. However, this study supports the idea that patch area is a relevant factor influencing biodiversity, also when applied to epiphytic lichens in peri-urban forest fragments in the Mediterranean area.

In agreement with [53,54], we found that the urban ecosystem and peri-urban areas provided a heterogeneous environment, leading to species rich community of macrolichen epiphytes.

This study proved peri-urban forest fragments provide important habitats for lichen diversity, and, in this framework, the sustainable environmental planning of the city should adequately consider the conservation of forest fragments, reducing their isolation by creating a specific ecological network and improving the habitat quality with detailed forest management.

## Figures and Tables

**Table 2:** Species list with occurrence (number of trees on which the species was detected) of epiphyte lichens detected in the *Quercus pubescens* forest patches in the study area.

Taxon	Occurrence
*Anaptychia ciliaris* (L.) Flot	19
*Blastenia ferruginea* (Huds.) A.Massal.	6
*Candelaria concolor* (Dicks.) Stein	2
*Candelariella xanthostigma* (Ach.) Lettau	19
*Collema flaccidum* (Ach.) Ach.	2
*Evernia prunastri* (L.) Ach.	9
*Flavoparmelia caperata* (L.) Hale	2
*Hyperphyscia adglutinata* (Flörke) H.Mayrhofer & Poelt	13
*Hypogymnia physodes* (L.) Nyl.	1
*Lecanora albella* (Pers.) Ach.	3
*Lecanora chlarotera* Nyl. subsp. *chlarotera*	14
*Lecidella elaeochroma* (Ach.) M.Choisyvar. *elaeochroma* f. *elaeochroma*	10
*Lepra albescens* (Huds.) Hafellner	5
*Lepra amara* (Ach.) Hafellner	15
*Lepraria finkii* (B. de Lesd.) R.C. Harris	8
*Melanelixia glabra* (Schaer.) O. Blanco, A. Crespo, Divakar, Essl., D. Hawksw. & Lumbsch	13
*Melanohalea elegantula* (Zahlbr.) O.Blanco, A.Crespo, Divakar, Essl., D.Hawksw. & Lumbsch	3
*Melanohalea exasperata* (De Not.) O.Blanco, A.Crespo, Divakar, Essl., D.Hawksw. & Lumbsch	13
*Myriolecis hagenii* (Ach.) Śliwa, Zhao Xin&Lumbsch	1
*Parmelia sulcata* Taylor	7
*Parmelina pastillifera* (Harm.) Hale	2
*Parmelina quercina* (Willd.) Hale	6
*Parmelina tiliacea* (Hoffm.) Hale	16
*Pectenia plumbea* (Lightf.) P. M. Jørg., L. Lindblom, Wedin & S. Ekman	2
*Pertusaria pertusa* (L.) Tuck. var. *pertusa*	12
*Physcia adscendens* (Fr.) H.Olivier	17
*Physcia aipolia* (Humb.) Fürnr.	8
*Physcia leptalea* (Ach.) DC.	1
*Physcia stellaris* (L.) Nyl.	3
*Physconia grisea* (Lam.) Poelt subsp. *grisea*	9
*Physconia perisidiosa* (Erichsen) Moberg	12
*Physconia venusta* (Ach.) Poelt	8
*Pleurosticta acetabulum* (Neck.) Elix&Lumbsch	26
*Ramalina farinacea* (L.) Ach.	4
*Ramalina fastigiata* (Pers.) Ach.	10
*Ramalina fraxinea* (L.) Ach.	8
*Xanthoria parietina*(L.) Th.Fr.	17

**Table 3:** Loadings of the most related variables (Spearman’s r > 0.3) with the two ordination axes of PCoA shown in Figure 3.

Variable	PC1	PC2
Patch area	0.21	−0.14
Slope	0.10	−0.50
Basal area	0.22	−0.01
Tree cover	−0.35	−0.01
Trunk diameter	0.17	−0.51

## Data Availability

Not applicable.
